# Quality Characteristics and In Vitro Digestibility of Starch Gel in White Noodles Prepared with Short-Chain Glucan Aggregates (SCGA)

**DOI:** 10.3390/gels11010006

**Published:** 2024-12-26

**Authors:** Jae-Geun Park, Sang-Jin Ye, Seon-Min Oh, Jae-Sung Shin, Ji-Eun Bae, Hyun-Wook Choi, Moo-Yeol Baik

**Affiliations:** 1 Department of Food Science and Biotechnology, Institute of Life Science and Resources, Graduate School of Biotechnology, Kyung Hee University, Yongin 17104, Republic of Korea; jaegyn@khu.ac.kr (J.-G.P.); lifesci2015@khu.ac.kr (S.-J.Y.); seonmin@kfri.re.kr (S.-M.O.); drumlover@naver.com (J.-S.S.); wise123@khu.ac.kr (J.-E.B.); 2 Food Processing Research Group, Korea Food Research Institute, Wanju-gun, Jeonju 55365, Republic of Korea; 3 Department of Food and Nutrition, Jeonju University, Jeonju 55069, Republic of Korea

**Keywords:** short-chain glucan aggregates (SCGA), noodle, resistant starch (RS), non-digestible maltodextrin (NMD), texture properties, in vitro digestibility of starch gel

## Abstract

Short-chain glucan aggregates (SCGA), a type of resistant starch (RS) Ⅲ, is produced by debranching amylopectin with pullulanase and inducing self-assembly. Despite its low digestibility and high RS content, SCGA has not been applied to real food systems, especially noodles. The objective of this study was to determine the feasibility of low-digestible noodles using SCGA and to evaluate their quality characteristics and in vitro digestibility of starch gel. SCGA-noodles (SN) were prepared by substituting 0, 10, 20, and 25% of wheat flour with SCGA, and non-digestible maltodextrin (NMD) replaced 7% of the flour for comparison. Adding SCGA increased L- and b-values of the dough, resulting in a brighter appearance, while the NMD increased a-values. The substitution with SCGA weakened the gluten network, reducing dough and texture properties. Notably, cooked SN25 broke immediately in the tensile test, indicating substitution up to 20% is feasible in noodles. NMD7 formed sticky dough and showed extensive elongation without breaking. SN20 and SN25 significantly increased RS content and decreased the estimated glycemic index (eGI) compared to the control. However, NMD7 did not significantly reduce in vitro starch digestibility. In conclusion, this research confirmed the potential of SCGA as a low-digestible ingredient for noodles and other food applications.

## 1. Introduction

With the increasing prevalence of obesity and diabetes worldwide, there is growing interest in nutrition and health [1,2]. As attention to health and weight management through diet increases, the demand for foods with a low glycemic index (GI) is also rising [3,4]. Consequently, there has been a growing focus on resistant starch (RS) in starch gel, which is known for its ability to lower the GI and provide nutritional benefits [5].

Starch is a primary component of the human diet and serves as a major energy source. It can be categorized into three types based on digestibility: rapidly digestible starch (RDS), slowly digestible starch (SDS), and resistant starch (RS). RDS is completely digested and converted into glucose within 20 min, SDS is digested more slowly in the small intestine taking up to 120 min, and RS is the fraction of starch that resists digestion and absorption in the small intestine [6]. RS exhibits similar physiological properties to dietary fiber, but it is known for its relatively high processing compatibility [7]. Based on its structure and properties, resistant starch can be classified into five types, as follows [8]:

RS1: Physically inaccessible starches found in whole or partially milled grains and seeds.

RS2: Native granular starches present in raw potatoes and unripe bananas.

RS3: Retrograded starches formed when starchy foods are cooled after cooking.

RS4: Chemically modified starches formed by cross-linking, etherization, or esterification.

RS5: Amylose–lipid complexes formed between amylose and specific lipids.

Among these, Shamai et al. [9] reported that RS3, which is produced in retrograded starch gel, has a relatively high melting temperature of 150 °C, indicating excellent thermal stability. The high thermal stability of RS3 suggests that its structure and properties can be maintained even when added to foods and cooked [10]. Short-chain glucan aggregates (SCGA), a type of RS3, are produced by debranching amylopectin with pullulanase and inducing self-assembly of glucans [11]. SCGA has thermo-reversibility and good thermal stability compared to native starch due to its structural properties, indicating that its structure can be preserved after cooking in foods and it may help to reduce digestibility, acting as a resistant starch [12]. It is anticipated to be useful as a novel low-digestible food ingredient due to its low in vitro digestibility and high RS content [13]. Despite its potential, no previous studies have applied SCGA to food systems. Therefore, this study aimed to incorporate SCGA into actual food products, particularly noodles, which are formed from a kind of starch gel.

Noodles are a kind of starch gel and are consumed worldwide, especially as a staple food in Asia [14]. There is also increasing demand for noodles with a lower GI and reduced digestibility [15,16]. However, existing low-digestible noodles, such as konjac and tofu noodles, may not appeal to all consumers due to their distinct texture and flavor [17]. Furthermore, konjac noodles require storage and distribution in water, which can be inconvenient. This has led to the need to develop novel low-digestible noodles based on wheat flour.

The objective of this study was to determine the feasibility of low-digestible noodles using SCGA and to investigate their quality characteristics and the in vitro digestibility of starch gel in cooked noodle. This study aimed to compare non-digestible maltodextrin (NMD), which is primarily used in the production of low-GI foods, with SCGA and to confirm new applications of SCGA in food industries.

## 2. Results and Discussion

### 2.1. Dough Mixing Properties by Mixolab

**Figure 1:**
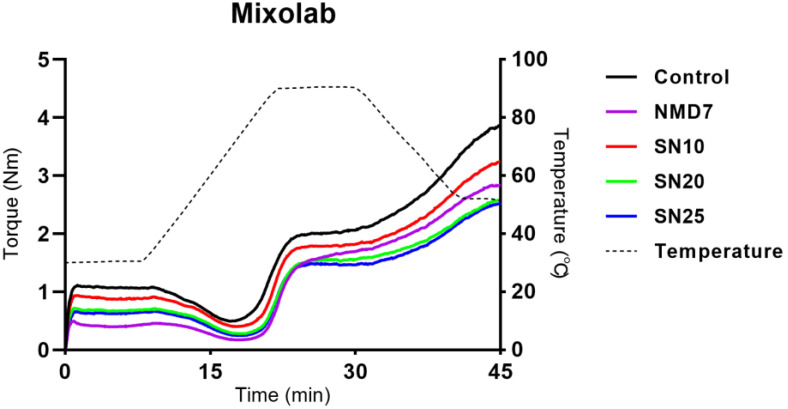
Mixolab profiles of the dough with SCGA and NMD.

Figure 1 shows the dough mixing properties measured by Mixolab based on the torque variation over time and temperature changes. The torque values represent the moment of force required for the rotation of the two blades inside Mixolab as water is added to form the dough [18]. A higher torque indicates that a greater force is needed to form the dough, implying a firmer dough [19]. The water content was kept constant across all samples to compare the dough properties, focusing on the characteristics of the additives, such as SCGA and NMD. Up to 8 min, the temperature was maintained at 30 °C under isothermal conditions, allowing for an assessment of the inherent stability of the dough without the influence of temperature changes [20]. Additionally, Schmiele et al. [21] have reported that the first stage of the Mixolab process is related to the farinograph and consistograph, which can assess the viscoelastic properties of the dough. Since noodles are not heated in their dough state to make starch gel but cooked after being shaped into noodle form, the discussion primarily focused on dough stability (C1) and protein weakening (C2).

As depicted in Figure 1, the dough with SCGA and NMD exhibited a decreased profile compared to the control. It was observed that the parameters decreased following the addition of SCGA and NMD (Table 1). Specifically, a significant reduction in C1 and C2 values was noted for SN20 and SN25, indicating a decrease in dough stability after the addition of more than 20% SCGA. This result can be attributed to the increased SCGA content, which led to a more fragile dough with weakened cohesion, thereby reducing the force required for dough formation. Interestingly, NMD7 showed the lowest torque profile before reaching the peak temperature. This may be attributed to the relatively high water-holding capacity of non-digestible maltodextrin (NMD), resulting in a less firm and stickier dough [22]. Excessive stickiness and elasticity can lead to lower processability and create a sticky and chewy texture, which is often undesirable in noodle production [23]. The results suggest that there is a limit to the acceptable level of NMD addition to the dough, considering its effect on dough texture and processability.

### 2.2. Color, Optimum Cooking Time, and Cooking Loss

Table 2 presents the color values of the dough just before cutting. The incorporation of SCGA significantly increased the L-value and decreased the b-value. It led to a noticeable increase in brightness compared to the control, which is preferred in white noodles [24]. Kang et al. [25] reported that the color of noodles was a key factor that consumers first assessed when evaluating the quality of noodles, and that noodles with a bright color were preferred. This suggests that SCGA may act as a color-enhancing agent, improving the brightness of the noodle dough. Conversely, the addition of NMD caused a significant increase in the a-value of the dough, indicating that it could negatively affect color quality of the noodles including NMD [26]. These findings emphasize the superior color-improving effects of SCGA in white noodle production.

Table 3 shows the optimum cooking time (OCT) and cooking loss of the cooked noodles for starch gelatinization in noodles. The reduction in the OCT and the increase in cooking loss, compared to the control, were attributed to the weakened gluten network as the wheat flour content decreased. These observations align with the findings of previous studies; Pu et al. [27] reported that when wheat flour was replaced with potato flour the OCT decreased while cooking loss increased. Deng et al. [28] observed a similar trend when noodles were formulated with modified potato starches. The reduction in the OCT was due to the lower wheat flour content. It weakened the protein network, making it more prone to damage during the cooking process. This damage facilitated easier water penetration into the noodle matrix, increasing the water content around starch molecules [29]. The rapid infiltration of water accelerated heat transfer from the surface to the inner white core of the noodles, promoting starch gelatinization and reducing the OCT. Furthermore, noodles with SCGA or NMD substituted for wheat flour exhibited reduced gluten network formation, which weakened the protein–starch binding. This led to increased leaching of starch and solid matter during cooking, thereby elevating the cooking loss. Notably, a higher proportion of SCGA significantly increased in cooking loss. This result is consistent with previous research indicating that cooking loss increases with greater additions of resistant starch in pasta [30]. The rise in the resistant starch content, such as SCGA, disrupts the protein network within the noodle matrix during cooking, resulting in higher cooking loss.

### 2.3. Texture Properties

**Figure 2:**
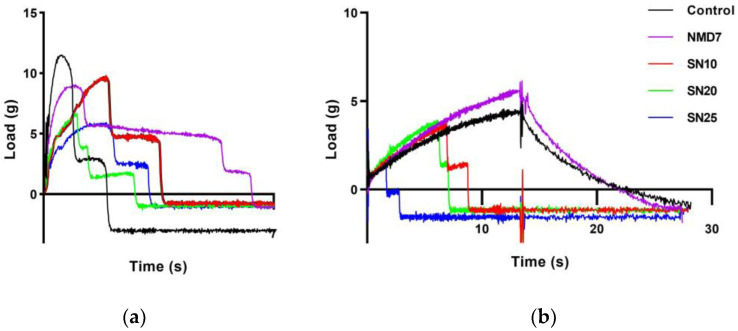
Tensile strength profiles of uncooked (**a**) and cooked noodles (**b**).

Table 4 presents the peak load values that indicate the maximum force that the uncooked and cooked noodle strands could withstand just before breaking. For the uncooked samples, a significant reduction in peak load was observed when SCGA content reached 20%, while NMD did not show significant differences. In the tensile profile of uncooked noodles, it is observed that the peak of NMD7 was elongated (Figure 2a). It was attributed to the high water-holding capacity of NMD, which caused the noodles to stretch before breaking. This finding aligned with the result from Section 2.1. Dough mixing properties by Mixolab, which reported excessive stickiness in both dough and noodles. It is interesting to note that “necking” phenomena rather than “delaminating” behavior was observed when the noodles were breaking, i.e., the cross-sectional area of the noodle began to decrease in this case.

For noodles with SCGA, the peak load after cooking significantly decreased across all samples. As shown in the dough properties analysis, the addition of SCGA weakened the cohesion within the dough, resulting in reduced tensile properties of the noodles. Notably, SN25 broke immediately upon the commencement of the tensile test (Figure 2b), which also explained the weakening of starch gel structure in cooked noodles. The sharp decline in peak load after fracture suggested that 25% substitution level of SCGA was not suitable for maintaining desirable noodle properties. In contrast, the control and NMD7 stretched continuously without breaking until the end of the test (Figure 2b). Notably, the peak load of NMD7 significantly increased after cooking. Similar to the dough properties, excessive stickiness and elasticity might contribute to an undesirable chewy texture [31].

Table 5 shows the Texture Profile Analysis (TPA) parameters. The addition of SCGA led to significant reductions in hardness, adhesiveness, chewiness, and springiness regardless of substitution levels. When the level exceeded 20%, cohesiveness and gumminess were significantly reduced. The addition of NMD showed either no significant difference from the control or a relative increase in chewiness. Both the tensile test and TPA results indicate a reduction in the gluten network and strength of starch gel. As the substitution level increased, the amount of available flour was reduced and this resulted in decreased gluten development and starch gelatinization. It ultimately weakened the textural properties of the noodles.

In conclusion, SCGA addition reduced the dough and noodle textural properties compared to the control. This effect was particularly notable at 25% substitution, where the noodles broke rapidly during the tensile test after cooking. The results suggest that 20% substitution is the optimal limit for maintaining noodle integrity. While the crumbly texture and fragility may be disadvantageous for noodle-making, these properties could have a positive impact in applications such as the confectionery sector or bread-making, where shortening is critical [32,33]. In confectionery applications, gluten development is often intentionally limited to achieve a crumblier texture, suggesting potential new applications for SCGA in promoting desirable textural properties [34].

### 2.4. In Vitro Digestibility of Starch Gel

Table 6 presents in vitro digestibility of starch gel in the cooked noodles. The RS content showed a significant increase in SN20 and SN25, suggesting that SCGA contributed to reduced digestibility and offered health benefits [35]. On the other hand, SN10 did not show a significant difference in RS compared to the control despite the addition of SCGA. This indicates that the substitution level in SN10 may not have been sufficient to significantly increase RS content or reduce digestibility. It was confirmed that the SCGA level of 20% or more improved RS content and digestibility. NMD did not exhibit any significant effects on the RS increase or digestibility reduction, possibly due to limitations on its substitution level resulting from its processing properties.

**Figure 3:**
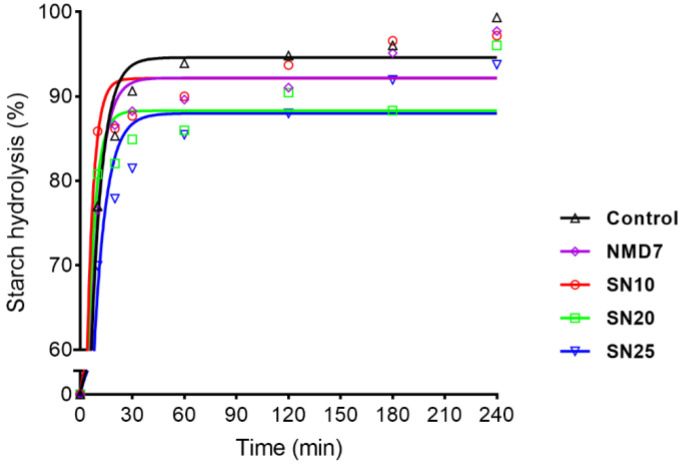
Hydrolysis curves of starch gel in the cooked noodles.

Figure 3 shows the hydrolysis rate of starch gel, depicted through the digestion curve, where starch hydrolysis (%) was plotted against time. AUC was calculated, and comparisons were made with white bread to derive the parameters shown in Table 7 [36]. No significant differences were observed for *C*_∞_ and k, but the eGI of SN20 and SN25 was significantly lower compared to the control. The reduction in eGI suggests that incorporating SCGA into the noodles effectively slows down the rate of digestion of starch gel, thereby lowering the glycemic response. It indicates that SCGA is beneficial for individuals managing hyperglycemia or diabetes [37].

Consequently, SN20 and SN25 exhibited a significant increase in RS content and reduction in eGI compared to the control, demonstrating its potential as a low-digestible ingredient. These findings have implications for the development of low-digestible foods aimed at glycemic control, especially in populations at risk for type 2 diabetes or metabolic syndrome [38]. The inclusion of SCGA as a novel low-digestible ingredient could not only improve the nutritional profile of commonly consumed foods like noodles, but also provide a means to enhance their health benefits while improving taste or texture, unlike konjac noodles.

## 3. Conclusions

This study aimed to explore the potential of using SCGA as a low-digestible ingredient in food products, particularly by assessing its effects on the quality and digestibility characteristics of noodles. The addition of SCGA resulted in increased L-values and decreased b-values in color analysis compared to the control, suggesting that SCGA could be useful as a color enhancer in products such as white noodles or other foods where a bright appearance is desirable. Due to the reduced gluten content, the dough characteristics and texture properties generally declined. Additionally, when replacing 25% of the wheat flour with SCGA, the tensile test indicated a low processability, suggesting that a substitution level of up to 20% is most suitable for noodle applications. SCGA supplementation significantly increased RS levels in SN20 and SN25 and lowered the eGI compared to the control, confirming SCGA’s potential as a low-digestible ingredient in starch gel foods.

When compared to NMD, it was observed that the addition of NMD increased the a-value of the dough, which could negatively affect the color characteristics of noodle products. The dough containing NMD was also found to be stickier due to its high water-holding capacity. Excessive stickiness beyond the optimal level can negatively affect the processing efficiency and may result in noodles with a tough texture. This contrasted with the SCGA dough and noodles, highlighting the differences between the two additives. In terms of in vitro digestibility of starch gel, no significant differences in RS and eGI were observed with the addition of NMD, due to the physical limitations on the amount that could be added.

The tendency of SCGA-enriched dough to break or crumble could be disadvantageous in noodle production but may have positive implications for baked goods or cookies, where a crumbly texture and shortening are desirable. In conclusion, this study established the characteristics of SCGA in noodle production and suggested additional potential applications in other product categories. The findings from this study contribute to the foundational understanding of SCGA applications and can support further research into its usage in various food products.

## 4. Materials and Methods

### 4.1. Materials

Wheat flour (13.6% moisture content, 9.5% protein, 1.4% lipid, and 0.4% ash) was purchased from Daehan Flour Mills Co. (Seoul, Republic of Korea). Waxy corn starch was obtained from Samyang Genex Co. (Seoul, Republic of Korea) and pullulanase (CAS number 9075-68-7, Promozyme D2; 1,350 NPUN/g, 1.15 g/mL) was purchased from Sigma-Aldrich Co. (St. Louis, MO, USA). Non-digestible maltodextrin was supplied by Daesang Co. (Seoul, Republic of Korea). Digestible and Resistant Starch Assay Kit was obtained from Megazyme International Ireland Ltd. (Wicklow, Ireland).

### 4.2. Preparation of Short-Chain Glucan Aggregates (SCGA)

SCGA was prepared according to the method of Oh, Park, Kim and Baik [11] with some modifications. Waxy corn starch (40 g, w.b.) was dispersed in 20 mM sodium acetate buffer (pH 5.0, 400 mL) and was then boiled with stirring for 30 min for gelatinization. After cooling down to 60 °C in a water bath for 30 min, pullulanase (60 μL/g starch) was added and the mixture was incubated at 60 °C for 12 h. The solution was stored at 4 °C for 12 h to induce self-assembly of short-chain glucans (SCG). After assembly, obtained SCGA was washed three times with distilled water using a centrifuge and dried at 40 °C overnight. It was ground using an SFM-C353NK electric mixer (Shinil Co., Seoul, Republic of Korea) and passed through a 100-mesh sieve.

### 4.3. Preparation of Noodles

The control noodle was prepared using wheat flour (100 g, w.b.), salt (2 g), and distilled water (38 mL) in an EGS Stand Mixer 600 (Sanlida Electrical Technology Co., Ltd., Shenzhen, China) [39]. After mixing the flour and salt, the mixture was placed into the bowl of the dough mixer. Half of the water was initially added to the mixture before kneading, and the remaining half was added after 1 min. The mixer was operated at speed 1 for the first 5 min and then at speed 2 for the next 5 min [40]. The dough was placed in a PE bag and developed in an EP-20 fermenter (Daeyung Bakery Machinery Co., Ltd., Seoul, Republic of Korea) at 25 °C for 30 min. After fermentation, the dough was sheeted 7 times to a thickness of 1.0 mm (4.8, 3.7, 3.1, 2.4, 1.8, 1.5, and 1.0 mm) and then cut to a width of 1.2 mm using an Atlas 150 pasta-making machine (Marcato, Campodarsego, Italy) [41]. SCGA-noodles were prepared by replacing 10%, 20%, and 25% of the wheat flour with SCGA and were designated as SN10, SN20, and SN25, respectively. Additionally, a comparison sample was produced by substituting 7% of the flour with non-digestible maltodextrin, designated as NMD7. The maximum addition levels were set at 25% and 7%, respectively, because the dough became crumbly when the SCGA content exceeded 25% and tearing occurred due to the stickiness of the dough when the NMD content exceeded 7%.

### 4.4. Dough Mixing Properties

The dough mixing properties of flour samples were determined using Mixolab (Chopin Technologies, Villeneuve-la-Garenne, France) with “Chopin+” protocol. The torque (Nm) was measured by Mixolab as a function of time and temperature during various stages of dough formation including mixing, heating, and cooling. The amount of each sample was calculated using Mixolab software (version 4.1.2.7), taking into account the moisture content of the samples and water absorption. The total mass of flour mixtures and distilled water was set to 75 g [42]. To observe the changes according to the content of wheat flour substitutes (SCGA or NMD), the water absorption was fixed at 51%, which corresponded to the control [43]. The Mixolab settings were as follows: mixing temperature of 30 °C for 8 min, heating at 4 °C/min up to 90 °C (15 min), cooling at 4 °C/min down to 50 °C (10 min), mixing speed of 80 rpm, and total analysis time of 45 min. The parameters (C1, C2, C3, C4, and C5) were obtained from the recorded curve.

### 4.5. Color Analysis

The color of the dough just before cutting was measured using a CR-20 colorimeter (Konica Minolta, Tokyo, Japan). Due to the thinness of the noodles, the surface of the dough was evaluated to ensure uniform color measurement. The instrument was calibrated using a white plate, and the color of each sample was measured seven times at random locations. The values of lightness (L*), redness (a*, positive), and yellowness (b*, positive) were recorded.

### 4.6. Optimum Cooking Time (OCT) and Cooking Loss

Optimum cooking time (OCT) and cooking loss were determined according to AACC method 66-50 with some modifications [44]. Each noodle sample was cooked in boiling distilled water on a hotplate. After 3 min of cooking, the noodle was taken out every 5 s and squeezed between two transparent glass plates. The OCT was determined by monitoring the time at which the white core in the noodle disappeared.

After cooking the samples of 5 cm length (5 g) in boiling water (300 mL) at OCT, the noodles were rinsed with distilled water (50 mL) in a Buchner funnel for 30 s [30]. The cooking and rinsing water were transferred to a beaker and dried in a dry oven at 105 °C. The weight of the residue was measured, and cooking loss was calculated based on the ratio of the noodle weight to the residue weight. Cooking loss was calculated as follows [45]:

$$Cooking\;loss\;(\%)=\frac{Weight\;of\;dried\;residue\;in\;cooking\;and\;rinsing\;water}{Weight\;of\;raw\;noodles}\times 100$$

### 4.7. Textural Properties of Noodles

#### 4.7.1. Tensile Test of Uncooked and Cooked Noodles

Tensile tests and textural properties were measured using a CTX texture analyzer (Ametek Brookfield, Middleborough, MA, USA). Tensile properties of uncooked and cooked noodles were determined with a TA-NTF probe and a 5 kg load cell as in the method reported by Sim et al. [46] with slight modification. The following settings were used: distance = 40 mm, pre-test speed = 1 mm/s, test speed = 3 mm/s, post-test speed = 3 mm/s, and trigger load = 0.5 g. To obtain cooked samples for the tensile test, 20 cm lengths of noodles were cooked at OCT, cooled with distilled water, and drained on a paper towel for 10 s before the measurement. The noodles were wrapped 2.5 times around each of the two knurled and horizontal columns of 1.8 cm diameter which were attached to the probe. The peak load was recorded by pulling the noodles vertically until just before they broke.

#### 4.7.2. Texture Profile Analysis (TPA) and Cooked Noodles

Textural properties of cooked noodles were determined using a TA7 probe and 5 kg load cell. The conditions were as follows: deformation = 70%, pre-test speed = 4 mm/s, test speed = 1 mm/s, post-test speed = 1 mm/s, and trigger load = 0.5 g [47]. The samples used in TPA were cut to a length of 5 cm, cooked, cooled, and drained. Five strands of noodles were placed on the platform and compressed vertically to 70% deformation of their original height according to AACC method 66-50 with some modifications [44]. Textural properties such as hardness, adhesiveness, cohesiveness, gumminess, chewiness, resilience, and springiness were obtained to assess the texture of the cooked noodles.

### 4.8. Measurement of In Vitro Digestibility of Starch Gel

#### 4.8.1. RDS, SDS, and RS Content

In vitro starch digestibility was measured using Megazyme Digestible and Resistant Starch Assay Kit, following the method described by McCleary et al. [48] with some modifications. The noodles were cooked and ground using a household blender for 10 s to simulate chewing. The sample (0.5 g) was wetted with 95% ethanol (0.5 mL) and dissolved in 50 mM sodium maleate buffer (17.5 mL, pH 6.0). Each tube was equilibrated in a water bath at 37 °C with stirring for 5 min. A quantity of 2.5 mL of PAA/AMG solution, which was prepared with pancreatic ɑ-amylase (CAS number 9000-90-2, PAA; 4 KU/5 mL), amyloglucosidase (CAS number 9032-08-0, AMG; 1.7 KU/5 mL), and 50 mM sodium maleate buffer, was added and the mixture was incubated at 37 °C with stirring. Aliquots (1.0 mL) were taken at 20, 120, and 240 min and mixed with 50 mM acetic acid solution (20 mL) to inactivate the enzyme. To determine total starch (TS), 4.0 mL of the aliquot at 240 min was taken and mixed with 95% ethanol (4.0 mL). After centrifugation, the supernatant was discarded, and the pellet was collected. It was washed twice with 50% aqueous ethanol (8.0 mL) and suspended in 1.7 M NaOH (2.0 mL) to dissolve the non-digestible starch fraction. The released glucose amount was quantified using a glucose oxidase/peroxidase (GOPOD) reagent. TS content was calculated by adding the total digestible starch content from 1.0 mL of the aliquot at 240 min and non-digestible starch content at 240 min. The RDS, SDS, and RS fractions were determined as follows [49]:

Rapidly digestible starch (RDS) = (G_20_ × F × 0.9 × 100)/W (1)

Slowly digestible starch (SDS) = ((G_120_ − G_20_) × F × 0.9 × 100)/W (2)

Resistant starch (RS) = TS − (RDS + SDS)(3)

where G = absorbance value of each time point, F = 100/GOPOD absorbance, and W = sample weight (mg), respectively.

#### 4.8.2. Estimated Glycemic Index (eGI)

The estimated glycemic index (eGI) of the cooked noodle was determined as in the method reported by Goñi, Garcia-Alonso and Saura-Calixto [36] with slight modifications. Aliquots were taken at 10, 20, 30, 60, 120, 180, and 240 min to determine a hydrolysis curve and calculate the starch hydrolysis rate. The kinetics of starch hydrolysis and eGI were calculated using a first-order equation: $C=C_{\infty }(1-e^{-kt})$, where *C* = the percentage of starch hydrolysis at time *t* (min), *C*_∞_ = the equilibrium concentration of hydrolyzed starch, and *k* = the rate constant. The area under the hydrolysis curve (AUC) was determined using the equation: $AUC=C_{\infty }t-\frac{C_{\infty }}{k}(1-e^{-kt})$. The hydrolysis index (HI) was calculated as the ratio of the AUC of each sample to the AUC of white bread, which was used as the reference food. The eGI was calculated based on the equation: eGI *=* 39.71 *+* 0.549 HI.

### 4.9. Statistical Analysis

Each experiment was performed at least three times. The results were expressed as means ± standard deviation. Starch hydrolysis curves and their parameters were calculated using GraphPad Prism software version 8.4.3 (GraphPad Prism Inc., San Diego, CA, USA). Statistical analysis was carried out using analysis of variance (ANOVA), followed by Tukey’s test with SAS software version 9.4 (SAS Institute Inc., Cary, NC, USA).

## Figures and Tables

**Table 1:** Mixolab parameters of the dough with SCGA and NMD.

	C1 (Nm)	C2 (Nm)	C3 (Nm)	C4 (Nm)	C5 (Nm)
Control	1.11 ± 0.03 ^a^*	0.49 ± 0.03 ^a^	1.84 ± 0.10 ^a^	2.06 ± 0.05 ^a^	3.84 ± 0.11 ^a^
NMD7	0.50 ± 0.02 ^d^	0.17 ± 0.01 ^d^	1.21 ± 0.03 ^b^	1.69 ± 0.09 ^bc^	2.82 ± 0.17 ^c^
SN10	0.95 ± 0.00 ^b^	0.40 ± 0.02 ^b^	1.66 ± 0.11 ^ab^	1.81 ± 0.09 ^b^	3.21 ± 0.15 ^b^
SN20	0.72 ± 0.08 ^c^	0.27 ± 0.02 ^c^	1.40 ± 0.32 ^ab^	1.54 ± 0.02 ^cd^	2.59 ± 0.04 ^c^
SN25	0.66 ± 0.05 ^c^	0.25 ± 0.01 ^c^	1.34 ± 0.12 ^b^	1.46 ± 0.05 ^d^	2.50 ± 0.13 ^c^

* Data with a different letter in the same column are significantly different (*p* < 0.05).

**Table 2:** Colors of uncooked dough with SCGA and NMD.

	L *	a *	b *
Control	83.36 ± 0.27 ^b^**	0.66 ± 0.09 ^b^	24.22 ± 0.19 ^a^
NMD7	84.16 ± 0.95 ^b^	0.92 ± 0.08 ^a^	24.96 ± 0.30 ^a^
SN10	88.94 ± 0.19 ^a^	0.46 ± 0.05 ^c^	21.22 ± 0.16 ^b^
SN20	88.74 ± 0.40 ^a^	0.28 ± 0.08 ^d^	19.98 ± 0.95 ^c^
SN25	89.24 ± 0.37 ^a^	0.24 ± 0.05 ^d^	18.82 ± 0.11 ^d^

* L = Lightness; a = redness; b = yellowness. ** Data with a different letter in the same column are significantly different (*p* < 0.05).

**Table 3:** Optimum cooking time and cooking loss of noodles.

	OCT	Cooking Loss (%)
Control	3 min 45 s	6.06 ± 0.34 ^d^*
NMD7	3 min 40 s	7.38 ± 0.07 ^c^
SN10	3 min 30 s	8.22 ± 0.25 ^c^
SN20	3 min 15 s	10.83 ± 0.37 ^b^
SN25	3 min 5 s	12.28 ± 0.33 ^c^

* Data with a different letter in the same column are significantly different (*p* < 0.05).

**Table 4:** Peak loads of uncooked and cooked noodles.

	Peak Load (g)	
	Uncooked	Cooked
Control	13.15 ± 3.46 ^a^*	5.40 ± 0.57 ^b^
NMD7	9.70 ± 0.52 ^ab^	7.37 ± 0.12 ^a^
SN10	10.15 ± 0.07 ^ab^	4.15 ± 0.07 ^c^
SN20	7.50 ± 0.44 ^b^	4.10 ± 0.14 ^c^
SN25	6.10 ± 0.28 ^b^	3.70 ± 0.28 ^c^

* Data with a different letter in the same column are significantly different (*p* < 0.05).

**Table 5:** TPA parameters of cooked noodles.

	Hardness (g)	Adhesiveness (mJ)	Cohesiveness	Gumminess (g)	Chewiness (mJ)	Resilience	Springiness (mm)
Control	45.10 ± 1.54 ^a^*	0.06 ± 0.01 ^a^	0.54 ± 0.03 ^ab^	24.30 ± 1.00 ^ab^	0.20 ± 0.01 ^b^	0.37 ± 0.03 ^a^	0.81 ± 0.02 ^a^
NMD7	46.60 ± 2.55 ^a^	0.06 ± 0.01 ^a^	0.61 ± 0.01 ^a^	28.30 ± 1.27 ^ab^	0.25 ± 0.00 ^a^	0.38 ± 0.00 ^a^	0.90 ± 0.04 ^a^
SN10	37.25 ± 1.34 ^b^	0.03 ± 0.00 ^b^	0.55 ± 0.03 ^ab^	19.40 ± 1.84 ^bc^	0.15 ± 0.02 ^c^	0.41 ± 0.01 ^a^	0.62 ± 0.01 ^b^
SN20	35.15 ± 0.49 ^b^	0.03 ± 0.00 ^b^	0.45 ± 0.06 ^b^	15.95 ± 2.19 ^c^	0.10 ± 0.01 ^d^	0.35 ± 0.04 ^a^	0.60 ± 0.01 ^b^
SN25	33.80 ± 0.42 ^b^	0.03 ± 0.01 ^b^	0.46 ± 0.01 ^b^	15.35 ± 0.07 ^c^	0.09 ± 0.01 ^d^	0.36 ± 0.04 ^a^	0.59 ± 0.05 ^b^

* Data with a different letter in the same column are significantly different (*p* < 0.05).

**Table 6:** In vitro digestibility of starch gel in the cooked noodles.

	RDS	SDS	RS
Control	85.34 ± 1.78 ^a^*	9.51 ± 1.92 ^a^	5.15 ± 0.14 ^b^
NMD7	86.58 ± 1.28 ^a^	8.55 ± 0.04 ^a^	4.87 ± 1.32 ^b^
SN10	86.19 ± 1.34 ^a^	7.51 ± 0.42 ^a^	6.30 ± 0.92 ^b^
SN20	82.08 ± 1.08 ^ab^	8.38 ± 0.42 ^a^	9.54 ± 0.65 ^a^
SN25	77.90 ± 0.89 ^b^	10.10 ± 0.04 ^a^	12.00 ± 0.15 ^a^

* Data with the different letter in the same column are significantly different (*p* < 0.05).

**Table 7:** Hydrolysis kinetics *** and eGI of starch gel in the cooked noodles.

	$C_{\infty }$ * (%)	k$\;*\;({min}^{-1}$)	HI *	eGI *
Control	94.63 ± 0.25 ^a **^	0.16 ± 0.02 ^a^	73.06 ± 0.14 ^a^	79.74 ± 0.07 ^a^
NMD7	92.20 ± 0.35 ^a^	0.17 ± 0.01 ^a^	71.40 ± 0.49 ^abc^	78.83 ± 0.27 ^abc^
SN10	92.16 ± 0.81 ^a^	0.26 ± 0.03 ^a^	72.37 ± 0.58 ^ab^	79.36 ± 0.32 ^ab^
SN20	88.28 ± 3.68 ^a^	0.25 ± 0.06 ^a^	68.94 ± 1.96 ^bc^	77.48 ± 1.08 ^bc^
SN25	88.01 ± 0.26 ^a^	0.14 ± 0.01 ^a^	67.99 ± 0.10 ^c^	76.96 ± 0.06 ^c^

* $C_{\infty }$ = equilibrium starch hydrolysis concentration; *k* = rate coefficients; HI = hydrolysis index; eGI = estimated glycemic index. ** Data with a different letter in the same column are significantly different (*p* < 0.05). *** The R^2^ values of control, NMD7, SN10, SN20, and SN25 for curve fittings were 0.9914, 0.9908, 0.9863, 0.9836, and 0.9822, respectively.

## Data Availability

The original contributions presented in this study are included in the article. Further inquiries can be directed to the corresponding authors.
